# Comparative Quality Evaluation of Selected Brands of Cefuroxime Axetil Tablets Marketed in the Greater Accra Region of Ghana

**DOI:** 10.1155/2021/6659995

**Published:** 2021-04-08

**Authors:** Christina Osei-Asare, Esther Eshun Oppong, Frederick William Akuffo Owusu, John Antwi Apenteng, Yvonne Ochesinda Alatu, Robert Sarpong

**Affiliations:** ^1^Department of Pharmaceutics, School of Pharmacy, Central University, Miotso, Ghana; ^2^Department of Pharmaceutics, School of Pharmacy and Pharmaceutical Sciences, University of Cape Coast, Cape Coast, Ghana; ^3^Department of Pharmaceutics, Faculty of Pharmacy and Pharmaceutical Sciences, Kwame Nkrumah University of Science and Technology, Kumasi, Ghana

## Abstract

The ever-growing commercialization of poor-quality and substandard medicines, especially anti-infectives characterized by inadequate postmarket surveillance by stakeholders remains a major global health challenge, particularly in developing countries, where antibiotic drug resistance and its repercussions on human health remain dominant. This research sought to evaluate the pharmaceutical quality of six randomly selected brands of cefuroxime axetil tablets (250 mg) marketed in the Greater Accra region of Ghana. The selected brands were coded and subjected to both compendial and noncompendial tests. Statistical analysis and model-independent parameter (similarity factor, *f*2) were employed in analyzing the dissolution profiles of all the brands. All brands including the reference brand conformed to the pharmacopeial specifications for both compendial and noncompendial tests, indicating that they were of good quality. However, there were significant variations (*p* < 0.05) in the disintegration time amongst the various brands. All the brands had ƒ2 values > 50 indicating similarity of their drug release profiles with the innovator. Hence, all the sampled cefuroxime axetil brands can be considered as pharmaceutical equivalents to the innovator drug. These brands can, therefore, be used as a substitute for the innovator drug by physicians to patients in cases of unaffordability or unavailability of the innovator brand.

## 1. Introduction

The global threat associated with the escalating promulgation of poor-quality medicinal products leaves much to be desired. Rather unfortunately, the rising market as a result of the global demand for pharmaceutical drugs (particularly anti-infectives), vaccines, and diagnostic kits has unfortunately created modern and shrewd opportunities for unprincipled companies, traders, and criminals [1]. To further this conversation, the global market has witnessed an unprecedented preponderance of extensive substandard and falsified drug varieties ranging from both costly and affordable anti-infectives, antimalarials, genitor-urinary, sex hormones, oncology, and analgesics of innovator or generic origin [2]. Thereby confirming to an extent, the situational data of the World Health Organization which suggest that one of every ten medicines in low- and middle-income countries is substandard or falsified [3]. Given the dangers associated with the use of substandard drugs, it is understandable that only a handful of studies from both developed and developing nations have demonstrated the economical and clinical dangers associated with the use of substandard or falsified (SF) drugs [3, 4]. The related economic upheavals of SF drug use translate to higher health expenditures for patients, healthcare systems, and government in the form of wasted resources, disability adjusted risks due to protracted sickness, poverty, reduced sales, and tax revenues [4, 5]. Notwithstanding, other studies have investigated some peculiar clinical repercussions related to SF drugs in terms of low treatment outcomes, aggravation of previous health conditions, adverse effects, toxicity-related effects, mortality, disease transmission, and development of antibiotic resistance [6–8].

In this context, Renschler et al. reported that an annual estimated death of 155,000 primarily in young children is associated with the use of SF anti-infective drugs such as antimalarial and antimicrobial medicines. Frimpong et al. reported that significant proportions of pediatric drugs in Ghana (Ashanti Region) are of poor quality. Hence, the need to tackle SF drugs holistically is urgent and critical [9]. Relatedly, another emerging risk as reported by recent studies is the lack of confidence and breached trust among SF drug users (with unmet therapeutic goals) toward legitimate drug use, the entire health system, and qualified healthcare professionals as well as some credible manufacturing industries [8, 10, 11]. This mistrust has refueled another emerging public health danger of self-diagnosing, self-prescribing, and misuse of drugs accessed from unethical open access global markets (e-commerce), thus complicating the already vicious cycle created [9, 12]. Most importantly, the documented devastating effects of antimicrobial resistance as a result of microbial selection and geographic spread associate with SF drug use makes the situation even more daunting in developing countries [8].

In Ghana, a majority of the handful reported studies of antimicrobial resistance have focused predominantly on the social perceptions and clinical factors arising from their indiscriminate use to the detriment of extensive postmarket quality evaluation research on the use of substandard and falsified drug brands [7, 13]. The latter which involves all the activities undertaken to obtain more data and product information after marketing authorization has been granted and made available for public use unfortunately remains under-reported in Ghana, whereas the majority of studies focus on biological activity assays of older drug molecules such as penicillins and their association with antimicrobial resistance; a few have reported on postmarket quality evaluations of some innovator and generic drug brands (local and foreign) of the same older generational antibiotics: amoxicillin, cotrimoxazole [14], ampicillin, chloramphenicol, trimethoprim/sulfamethoxazole [15], kanamycin [16], metronidazole, ciprofloxacin, erythromycin, sulphamethoxazole, and trimethoprim [17]. Consequently, these reported studies have confirmed limited potency of the majority and their aftermath role in the development of antibiotic resistant strains, thereby limiting their extensive use currently in healthcare practice aiming at high treatment outcomes.

Given the potential dangers of antibiotic resistance regarding the use of substandard antibiotics and the current abysmal postmarket quality surveillance reports on the newer, effective and more expensive molecules of antibiotics such as cephalosporins (cefuroxime axetil axetil), the call for more stringent postmarket quality surveillance is timely and tenable. Postmarket quality evaluation of pharmaceuticals cannot be underestimated since it comprises of all activities undertaken as a means of obtaining more data and information about a product after it has been granted marketing authorization and made available for public use [18].

Cefuroxime axetil, a broad-spectrum second-generation cephalosporin antibiotic, is currently included in Ghana's standard treatment guidelines as one of the most effective antibiotic options for the treatment of a wide range of bacterial infections with proven efficacy. Its usefulness in Ghana is marked by a high proliferation of various drug brands (innovator and generic), either as dry powder for constitution or as tablets in varying doses (125 mg, 250 mg, and 500 mg), for both the young and elderly. Most probably, it may be considered preferable to the older molecules in terms of better treatment outcomes, with lesser tendency of development antimicrobial resistance in contrast to the previous older generation molecules [19]. Unsurprisingly, it ranked 5th among other 37 different classes of antibiotics in a retrospective study conducted at the Tamale Teaching Hospital in Ghana and 4th out of the top five antibiotics used at the Korle-Bu Teaching Hospital, the leading national referral hospital in Ghana [20]. This signifies its preference to other antibiotics in terms of efficacy. However, its potential risk of causing antimicrobial resistance was demonstrated by being ranked first (5.6%) in terms of all antibiotics (frequency of administration) errors recorded [7]. Coupled with the paucity of data supporting its postmarket quality surveillance in Ghana, there remains greater need for a more rigorous approach in monitoring its integrity during its entire shelf life and clinical use. In effect, this study serves as one of the maiden postmarket *in vitro* quality assessments of randomly selected popular brands of cefuroxime axetil (250 mg) tablet brands commonly marketed in Ghana and assesses the possibility of interchanging brands based on their similarity and difference factors. Consequently, it will provide an evidence-based postmarket product quality information on the investigated brands, reverberating the need for more field monitoring efforts to fight against the global threat of low-quality drugs and the associated dangers of antibiotic resistance.

## 2. Materials and Methods

### 2.1. Materials and Equipment

Cefuroxime axetil, monosodium phosphate (NaH_2_PO_4_), disodium phosphate (Na_2_HPO_4_), and methanol were obtained from Ernest chemists manufacturing outlet (Tema, Ghana). Other materials and equipment used included porcelain mortar and pestle, volumetric flasks (50 ml, 100 ml), measuring cylinders, dropper, conical flask, and filter papers. All reagents used were of analytical grade. The equipment used in this study included OHAUS Corp AR3130E electronic weighing balance (Switzerland), CS-I 02343 friabilator (Dongguan Walter Technology Co., Ltd., China), RC-1 0CI81 dissolution apparatus (Jinhu Minsheng Pharmaceutical Machinery Co., Ltd., China), UV spectrophotometer (Drawell-DU8000200, Shanghai Drawell Scientific, China), BJ-I OLCT2 disintegration tester (Minhua Pharmaceutical Machinery Co., Ltd, China), Copley TH3 hardness tester (UK), and Jenco 6175 pH meter (San Diego, USA).

## 3. Methods

### 3.1. Sampling of Cefuroxime Axetil Tablets

Six different brands of 250 mg cefuroxime axetil tablets were randomly purchased from various registered retail pharmacy outlets in Tema in the Greater Accra region of Ghana and coded as A, B, C, D, E, and F. The six drugs sampled and coded included an innovator brand and brands from the United Kingdom, India, and also a locally manufactured brand from Ghana.

### 3.2. Uniformity of Weight Test

Twenty tablets of each of the six different brands were used to conduct the uniformity of weight test. Each of the twenty tablets was weighed individually and recorded. The average tablet weight was calculated for, as well, determination of the percentage deviation from the mean for each tablet brand [21].

### 3.3. Friability Test

Ten tablets of each brand were selected randomly, weighed, and recorded. The tablets were placed in the friabilator, one brand at a time, to run at 25 revolutions per minute for 4 minutes. The tablets were then removed, cleared from loose dusts, and observed for any capping. The tablets were weighed and their percentage weight loss calculated [21].

### 3.4. Disintegration Test

Six tablets were randomly selected from each brand for this test. The selected tablets were placed individually in each of the six cylindrical tubes of the basket rack of the disintegration apparatus. The bottom of the basket rack was positioned such that it was at least 15 mm below the surface of the distilled water and the experiment was conducted at 37°C ± 2. The disintegration time was taken to be the time no granule of any tablet was left on the mesh. Three determinations were done and the mean disintegration time was determined for each brand [21–23].

### 3.5. Hardness Test

Ten tablets were selected at random from each brand to perform this test. A tablet was placed between the spindle and anvil of the Copley hardness tester and the calibrated scale adjusted to zero. The spindle was turned until the tablet broke apart. This force was recorded in Newtons. A mean hardness with standard deviation was calculated for each brand [21, 23].

### 3.6. Assay

Twenty tablets of each sampled brand of cefuroxime axetil were weighed and powdered. A quantity of the crushed powder equivalent to 0.25 g of cefuroxime axetil was shaken with 70 ml of methanol for 15 minutes, diluted to 100 ml with methanol, and filtered with Whatman filter paper (no. 5). Ten milliliters of the filtrate was diluted to 100 ml with methanol, and after serial dilutions, the absorbance of the resulting solution was measured at a wavelength of maximum absorption 276 nm. The content of cefuroxime axetil was then calculated using the previously determined calibration curve (*y* = 0.09*x* + 0.064, *r*^2^ = 0.997) [22–24].

### 3.7. Dissolution

Six randomly selected tablets from each brand were used for this test. Dissolution studies were carried out using the USP-XXII dissolution apparatus-2, paddle-type (RC-1 0CI81), at a rotational speed of 100 rpm at 37 ± 0.5°C. Each tablet was placed in a dissolution vessel and the dissolution media used was 900 mL of 0.1 M HCl. At fixed time intervals (10, 15, 30, 45, and 60 minutes), 10 ml of the dissolution media was withdrawn and the same quantity replaced with 0.1 M HCL to maintain sink conditions. The withdrawn samples were filtered, serially diluted, and spectrophotometrically analyzed at 276 nm on Drawell-DU8000200 UV spectrophotometer. The absorbance obtained was inserted into a previously determined calibration curve to obtain the amount of drug released. A plot of the cumulative drug release against time was then used to obtain the dissolution profile. This was done for all the sampled brands [22, 24].

### 3.8. Dissolution Data Analysis

Model-ndependent approach was used in investigating the similarity factor (*f*2) between the various brands and the innovator [25–27].

### 3.9. Data Analysis

The results are presented as the mean ± standard deviation. Data were analyzed with Excel (Windows version 8) and Graph Pad Prism version 5.00 for Windows (Graph Pad Software, San Diego California, USA, http://www.graphpad.com), respectively.

## 4. Results and Discussion

### 4.1. Sampled Brands of Cefuroxime Axetil

Table 1 gives a summary of information obtained on the sampled products. All samples selected were coded and analysis was performed without the packaging. Only codes were used to identify the various samples and thus the possibility of any bias was avoided. All the samples purchased had at least 6 months left on the shelf life and all analytical procedures were carried out before product expiration. The wide disparity in cost between the innovator and the generics may affect the ability of patients to comply with treatment when the innovator drug is prescribed for them.

### 4.2. Weight Uniformity of Tablets

Uniformity of weight is one of the tests which is conducted to ensure constant and uniform dosing among tablets within a batch and helps to prevent the incidence of underdosing or overdosing. Consequently, the weight variation of the individual tablet is a valid indication of the variation corresponding to the drug content [28]. According to the British pharmacopeia specifications for uniformity of weight, all the brands passed the uniformity of weight test as shown in Table 2. The brands passing the test could be attributed to good flow properties of granules and regular movement of the lower punch resulting in uniform die volume and hence uniform distribution of weight of the tablets [28]. In this study, the positive weight uniformity tests for the different brands at varied times of their shelf life during this postmarket analysis suggests compliance of the various manufacturers to good manufacturing practices (GMP) as demonstrated in the consistency of tablet weights and content uniformity of the formulation, irrespective of variations in excipients or active drug used as well as different tablet die sizes and fill volumes [29].

### 4.3. Hardness and Friability of Sampled Brands

Friability and hardness tests help to assess the ability of a tablet to withstand abrasion that is usually associated with packaging, handling, and transportation. These tablet properties are mainly influenced by the nature and amount of the binder usually used and force of compression [22]. From the results obtained (Table 2), all the brands passed the friability and hardness test indicating that the force of compression, type of binder, and its concentration were adequate [21, 29]. Aesthetically, they will encourage consumer acceptance of drug product and encourage drug administration compliance.

### 4.4. Uniformity of Dimensions of Sampled Brands

Dimensional tests are rapid in-process tests which are done to determine and confirm consistency in the size of tablets being produced. These tests help in confirming the weight uniformity of the manufactured tablets. All the sampled brands had their dimensions (length, thickness, and width) within the stipulated range of ±5% (Table 2) and hence conformed to specifications. These indicate that there was consistency in the manufacturing variables such as compression force, filling of the die cavity, lower and upper punch movements, and die volume [22, 27].

### 4.5. Disintegration Time

**Figure 1:**
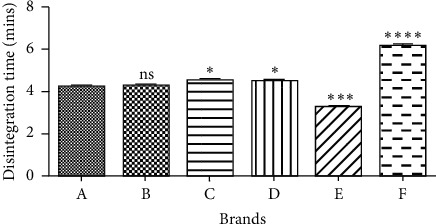
Statistical analysis on the disintegration time of sampled brands using *T*-test. Values are mean ± SD. *p* > 0.05 not significant (ns), *p* ≤ 0.05 significant (*∗*), *p* ≤ 0.001 significant (∗∗*∗*), and *p* ≤ 0.0001 significant (∗∗∗*∗*).

Disintegration is the process whereby tablets break down into smaller particles and is the first crucial step toward dissolution. For film coated tablets, disintegration of tablets into smaller fragments should not exceed thirty minutes [2]. All the sampled brands passed the disintegration test (Table 2). Sample E had the shortest disintegration time, followed by samples A, B, D, C, and finally F, respectively. The tablets disintegrating within time means that they were adequately compressed during manufacture with the right amount of the necessary excipients. However, the different disintegration times between the different brands could be because of different binders or disintegrants used in manufacturing.  Figure 1 presents a statistical representation of the different disintegration times of the six brands. It can be observed that brands A and B are statistically similar, since they both had similar disintegration time.

### 4.6. Drug Content of Sampled Brands

The purpose of this test is to determine the amount of active pharmaceutical ingredients (API) in each tablet of the six brands of cefuroxime axetil. The drug content of cefuroxime axetil is expected to be within 95–110% of the labelled claim [23]. Based on the results obtained (Table 2), all the sampled brands passed this test and could be attributed to good manufacturing practices during the preparation of the tablets. This implies that all the brands contain the required amount of the API and hence will not produce an underdose or overdose in the patient.

### 4.7. *In Vitro* Dissolution Profile of Sampled Brands

**Figure 2:**
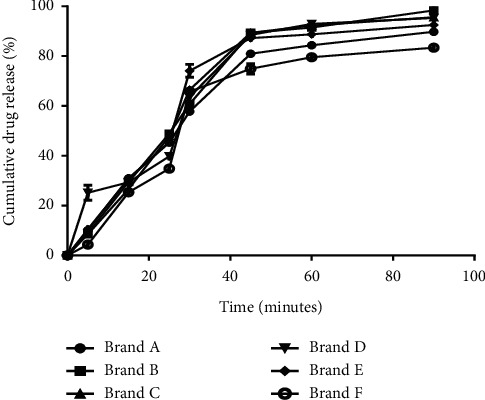
Dissolution profile of sampled brands of cefuroxime axetil (250 mg) tablets.

Dissolution is defined as the rate of mass transfer from a solid surface into a dissolution medium under standardized conditions of liquid/solid interface, temperature, and solvent composition. Currently, dissolution test is used as an in vitro bioequivalence test, for figuring out dissolution profile in general and for profile comparison and establishing the similarity of pharmaceutical dosage form [25].  Figure 2 shows the *in vitro* drug release profiles of the sampled brands of cefuroxime axetil. According to the USP, after 45 minutes of carrying out the dissolution test, not less than 75% of the active ingredient should be released from the tablet. Based on the USP dissolution requirement, all the brands passed the dissolution test and hence will release the expected amount of drug for the needed therapeutic action.

### 4.8. Dissolution Profile Comparison

In dissolution comparison, especially to assure similarity in product performance between an innovator and a generic drug, regulatory bodies are interested in knowing how similar the two curves are and this can be deduced using the similarity (*f*2) factor. Two dissolution profiles are found similar and considered bioequivalent if *f*2 value is between 50 and 100. All the brands had their *f*2 values falling within the standard range (Table 3) and therefore their dissolution profile is similar to that of the reference drug product and can be used interchangeably [27, 31, 32]. Furthermore, according to the FDA classification (FDA Orange Book), these pharmaceutical equivalent brands can also be regarded as therapeutic equivalents [33]. They can therefore be conveniently substituted with the innovator drug or with each other on the basis of availability, affordability, or patient tolerability.

## 5. Conclusion

The pharmaceutical quality of six brands of cefuroxime axetil tablets (250 mg) on the Ghanaian market was successfully evaluated. All the sampled brands passed the pharmacopeia and nonpharmacopeia tests conducted. None of the evaluated brands could be considered as substandard. All sampled brands of cefuroxime axetil brands in this study could be considered as pharmaceutical equivalents or interchangeable to the innovator drug based on the *in vitro* comparison of their dissolution profiles with the model-independent fit factor.

## Figures and Tables

**Table 1:** Brands of cefuroxime axetil (250 mg) tablets sampled.

Samples	Manufacture date	Expiry date	Batch number	Cost per pack (Ghana cedis)
Innovator (A)	June, 2017	June, 2020	C817624	75.0
B	February, 2018	February, 2021	JA9A	40.5
C	November, 2017	October, 2020	T17037	35.0
D	August, 2017	July, 2020	STR16A011	29.0
E	April, 2017	March, 2020	570022	25.5
F	October, 2017	October, 2020	0610T	32.5

**Table 2:** Evaluated physicochemical properties of sampled brands of cefuroxime axetil (250 mg) tablets.

Sample code	Average weight (g), *n* = 20	(%) Friability, *n* = 10	Uniformity of width (mm), *n* = 10	Disintegration time, *n* = 6	Hardness (kg/f), *n* = 10	Drug content (%)
A	0.450 ± 3.29	0.22	6.7 ± 0.02	4.21	8.9 ± 0.03	99.41
B	0.456 ± 3.61	0.22	6.7 ± 0.03	4.29	9.2 ± 0.01	99.17
C	0.570 ± 3.84	0.18	6.8 ± 0.01	4.55	10.4 ± 0.06	98.52
D	0.479 ± 4.36	0.41	6.7 ± 0.04	4.49	9.4 ± 0.02	98.14
E	0.431 ± 3.44	0.47	7.7 ± 0.01	3.30	8.2 ± 0.03	97.31
F	0.551 ± 2.87	0.18	7.2 ± 0.01	6.16	7.9 ± 0.01	98.05

**Table 3:** Similarity factor (*f*2) analysis between innovator brand (A) and sampled brands.

Brand	Similarity factor (*f*2)	Comment
B	62.3	Similar
C	59.4	Similar
D	53.0	Similar
E	61.0	Similar
F	59.0	Similar

## Data Availability

The data used to support the findings of this study are included in the article and also available from the corresponding author upon request.
